# Color Change in Commercial Resin Composites with Different Photoinitiators

**DOI:** 10.3390/bioengineering12101047

**Published:** 2025-09-28

**Authors:** Feng Gao, David W. Berzins

**Affiliations:** 1 School of Dentistry, Marquette University, Milwaukee, WI 53233, USA; 2 College of Dental Medicine-IL, Midwestern University, Downers Grove, IL 60515, USA

**Keywords:** aging, color change, commercial resin composites, photoinitiator, polymerization, TPO

## Abstract

The yellowing effect of camphorquinone (CQ) has led manufacturers to add alternative initiators into resin composites (RCs) to reduce the amount of CQ used. The aim of this study was to investigate the color change in commercial RCs with alternative photoinitiators besides CQ. Color change upon polymerization and aging in air and artificial saliva for up to 3 months was tested for seven commercial RCs (traditional and bulk-fill) with either CQ only or CQ and additional photoinitiators (CQ+). Color measurements were obtained with a spectrophotometer. Color change (ΔE) was calculated using the CIELab and CIEDE2000 formulae. ANOVA and a post hoc SNK test were conducted for statistical analysis. Upon polymerization, the ΔE of CQ+ was greater than that of CQ only, except in the case of dual-cure HyperFIL. The storage conditions did not affect the color change within 24 h for either air or artificial saliva, whereas they did have an influence on color stability when RCs were aged for 1 month and 3 months. The color changes in the RCs aged in artificial saliva were considered clinically acceptable for all RCs tested except HyperFIL. Additional photoinitiator systems tended to result in a greater color change upon polymerization but did not affect color change upon aging. During shade selection, especially when additional photoinitiators besides CQ are used, a guide reflecting the color after polymerization should be used.

## 1. Introduction

Since the early 1970s, dental resin composites (RCs) have been the material of choice for direct esthetic anterior and posterior restorations. Dental RCs serve as a more esthetic material to use over dental amalgam. Initiators are needed for dental RCs to start the polymerization process. In light-cured RCs, photoinitiators in the soft raw material are activated by the curing light, and they start the cascade reaction of polymerization, which enhances the mechanical properties [1].

Camphorquinone (CQ) is the most common photoinitiator used in light-cured dental RCs and adhesives [2,3]. This yellow agent is activated by absorbing external blue light. Although CQ in conjunction with a tertiary amine has traditionally been used in dental RCs, the intensive yellow color of CQ has limited its use, particularly in extra-white shades of RCs [4]. Other initiators, such as trimethylbenzoyl diphenylphosphine oxide (TPO) [5] and Ivocerin^®^, have been introduced as alternatives [6]. The absorption spectrum of TPO is situated more toward the UV spectrum (380–425 nm), with the peak at 400 nm [6,7,8]. Currently, TPO is the most often used additional photoinitiator in commercial dental RCs in the U.S. market. TPO’s wide adoption is related to its high efficiency and less-yellow profile [9]. TPO is completely colorless after light curing [10]. Ivocerin^®^ is a newly synthesized Germanium-based photoinitiator. The absorption peak of Ivocerin^®^ is around 418 nm [8]. According to the manufacturer, Ivocerin^®^ has an initial yellow tinge and requires a considerably lower concentration to achieve similar properties as standard photoinitiators.

The addition of the alternative photoinitiators lowers the content of CQ and has been suggested to overcome the esthetic issue arising due to the color of CQ [11,12]. However, the majority of the studies on color change in RCs due to photoinitiators were conducted on experimental RCs, because the chemical compositions to be studied, including photoinitiators, can be well controlled in these experimental formulations. The data on the color stability of commercially available RCs with additional photoinitiators besides CQ (CQ+) are insufficient to show whether or not such an approach is beneficial. The aim of this study was to investigate the color change in commercial traditional and bulk-fill dental RCs with alternative photoinitiator systems upon polymerization/curing and aging in artificial saliva and room air. The null hypothesis is that the color change after curing or aging in commercial RCs with CQ+ is not different from that observed in commercial RCs with CQ only.

## 2. Materials and Methods

### 2.1. Resin Composites

Seven commercial RCs, including traditional and bulk-fill RCs with and without additional photoinitiators besides CQ, were tested (Table 1). All materials were purchased, except AELITE, which was received as a research gift from the manufacturer. When available, shade A2 was used. If A2 was not available, the closest shade to A2 was used. Most of the manufacturers do not disclose what the exact additional photoinitiators are, because the information is considered to be proprietary. However, such information can be inferred from the literature (Table 1) [4,5,13,14,15].

### 2.2. Light Curing

**Figure 1:**
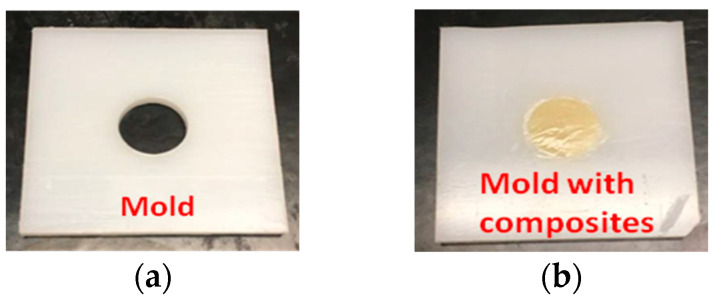
Mold used with (**a**) and without (**b**) RCs.

Sixteen samples for each material were prepared with the Teflon disc molds (9 mm diameter × 2 mm thickness) (Figure 1a,b). A 5 kg weight was kept on top of the RCs for 3 min to obtain a uniform surface. A clear plastic film was used to cover the RCs before the weight was applied and removed after light cure. RCs were light-cured for 40 s with a quartz–tungsten–halogen light-curing unit (Optilux 501, sds Kerr Sybron dental specialties, Danbury, CT, USA). The light intensity was 500 mW/cm^2^. Halogen light was used because of its broad spectrum of wavelengths.

### 2.3. Aging

A simple phosphate-buffered saline [16] was chosen to be the artificial saliva in this study to avoid the use of certain organic components that may be more likely to alter the color appearance of the RCs. Samples were randomly assigned into two groups (*n* = 8/group) after light cure: one group was stored in artificial saliva, and the other group was stored in air. Different storage conditions allow for differentiation of any color changes due to time from those due to the solution. Artificial saliva was used to simulate oral conditions, while room air was used to study color changes solely due to the material. Both groups were kept at 37 °C in an incubator, with each sample in an individual container to avoid any cross-contamination. All experiments were conducted in the same laboratory room with a controlled environment.

### 2.4. Color Measurements

**Figure 2:**
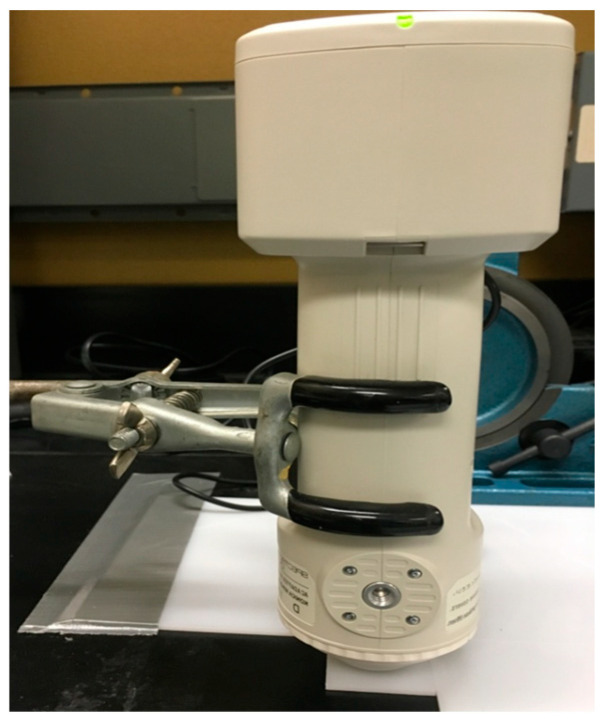
Color measurements were obtained with a spectrophotometer in a fixed position.

L*, a* and b* values were obtained with a spectrophotometer (Konica Minolta CM-700D, Ramsey, NJ, USA) right before light curing, soon after light curing and at different time points after aging for all samples. The spectrophotometer was kept in a fixed position as shown in Figure 2. The time points after aging were 1 h, 2 h, 3 h, 4 h, 5 h, 6 h, 1 day, 1 month and 3 months. At each time point, three measurements were taken, and the average of them was used. Samples were covered with a clear plastic film when color was measured, right after the weight was removed before light curing. Two color measurements were taken soon after light curing (less than 10 s), one with the clear plastic film covering the RCs as it was before light curing and another after removal of the film. Measurements from 1 h to 3 months were taken without the film.

### 2.5. Color Change

Color change was calculated using the CIELab formula (ΔE*_ab_) and the CIEDE2000 formula (ΔE_00_) as shown in Equations (1) and (2) [17,18]. K_L_, K_C_ and K_H_ were set as 1. Color change between the two measurements at “soon after light curing” with and without the film was calculated to ensure no color change was registered by the use of the clear plastic film. Calculations were conducted using the time points (1) between “before light curing” and “soon after light curing” to study the effect of polymerization and (2) between “soon after light curing” and each time point after aging to study the effect of aging.

(1) $$\Delta {E*}_{\mathrm{ab}}=\sqrt{(\Delta {L*)}^{2}+(\Delta {a*)}^{2}+(\Delta {b*)}^{2}}$$

(2) $$\Delta E_{00}=\sqrt{\left(\frac{\Delta L^{\prime }}{K_{L}S_{L}}{)}^{2}+(\frac{\Delta C^{\prime }}{K_{C}S_{C}}{)}^{2}+(\frac{\Delta H^{\prime }}{K_{H}S_{H}}{)}^{2}+R_{T}(\frac{\Delta C^{\prime }}{K_{C}S_{C}})(\frac{\Delta H^{\prime }}{K_{H}S_{H}}\right)}$$

### 2.6. Statistics

To determine the sample size, α and β errors of 5% and 20% were set, respectively. Effect size was calculated using data from pilot studies. For aging at 1 day, 1 month and 3 months, two-way ANOVA was conducted with photoinitiators (CQ or CQ+) and aging methods (in air or in artificial saliva) as factors. In order to study the effect of brand in more detail, one-way ANOVA with the resin composite brand as the factor was performed for the color changes upon polymerization and aging at 1 month and 3 months. When the data failed the normality test or equal variances test, ANOVA on rank test was conducted instead. The Tukey test was used as post hoc analysis. α level was set as 0.05 (significant when *p* < 0.05).

## 3. Results

### 3.1. Color Change upon Polymerization

**Figure 3:**
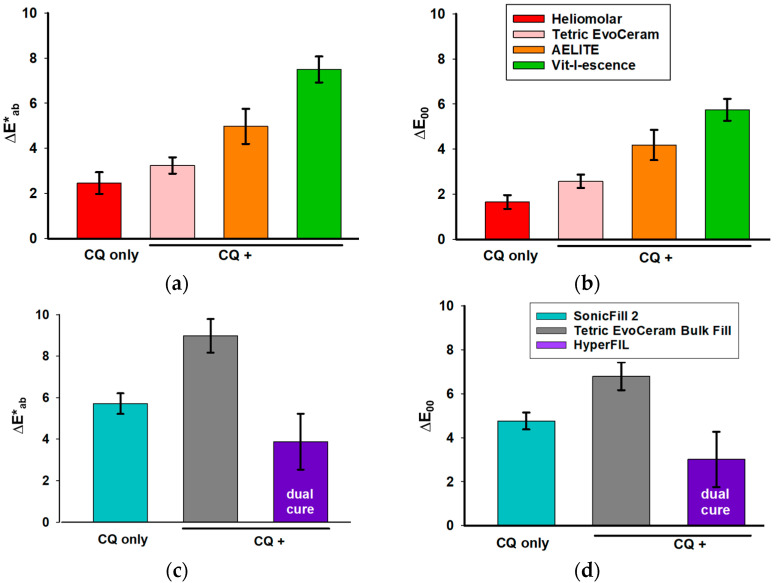
Color changes in traditional (**a**,**b**) and bulk-fill (**c**,**d**) RCs upon polymerization are shown. ∆E (delta E) was calculated by the CIELab formula (**a**,**c**) and CIEDE2000 formula (**b**,**d**). *N* is 16 for each group. Bars present mean and standard deviation. One-way ANOVA (**a**,**b**) or ANOVA on rank test (**c**,**d**) was conducted. All groups are significantly different from each other (**a**–**d**).

**Figure 4:**
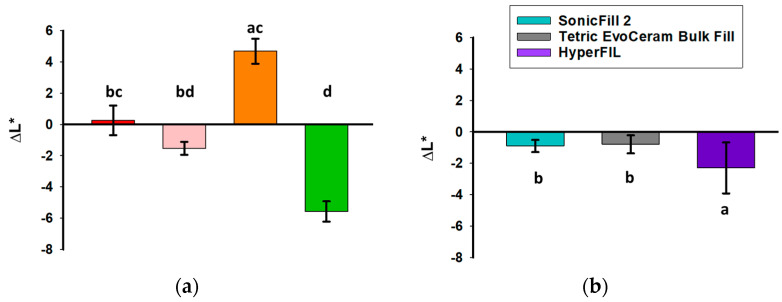

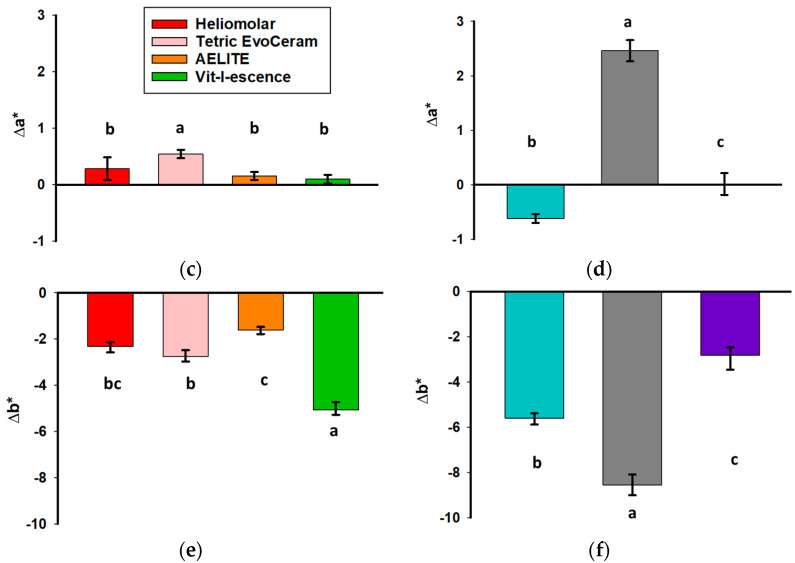
ΔL* (**a**,**b**), Δa* (**c**,**d**) and Δb* (**e**,**f**) of traditional (**a**–**c**) and bulk-fill (**d**–**f**) RCs upon polymerization calculated with the CIELab formula are shown. ∆L* (delta L) is the difference in L*. ∆a* (delta a) is the difference in a*. ∆b* (delta b) is the difference in b*. *N* is 16 for each group. Bars show the mean and standard deviation. One-way ANOVA (**f**) or ANOVA on rank test (**a**–**e**) was conducted. Significance is shown by bars labeled with different letters.

The L*, a* and b* of the uncured resin composites are shown in Table 2. Color change upon polymerization was measured soon after light curing (within 10 s). For the traditional RCs, those with additional photoinitiators besides CQ had a greater color change than the RCs with CQ only (Figure 3a,b). For the bulk-fill RCs, Tetric EvoCeram (CQ with additional photoinitiators) had a greater color change upon polymerization compared to SonicFill 2, the bulk-fill RC with CQ only; whereas HyperFIL (dual cure with CQ and additional photoinitiators) had less of a color change upon polymerization than SonicFill 2 (Figure 3c,d). Evaluating the color changes upon polymerization via the CIELab formula (Figure 3a,c) and CIEDE2000 formula (Figure 3b,d) resulted in the same significance groupings among RCs, though the values of ΔE_00_ are less than those of ΔE*_ab_ (Figure 3). The ΔL* values between before curing and soon after curing did not show a consistent pattern (Figure 4a,b). However, the Δb* values of all the RCs were less than zero, meaning that all the RCs tested became less yellow after light curing (Figure 4e,f). ∆a* was positive for all but one product (SonicFill 2, Figure 4d), suggesting that the RCs tended to become more red than green (Figure 4c,d).

### 3.2. Color Change upon Aging

**Figure 5:**
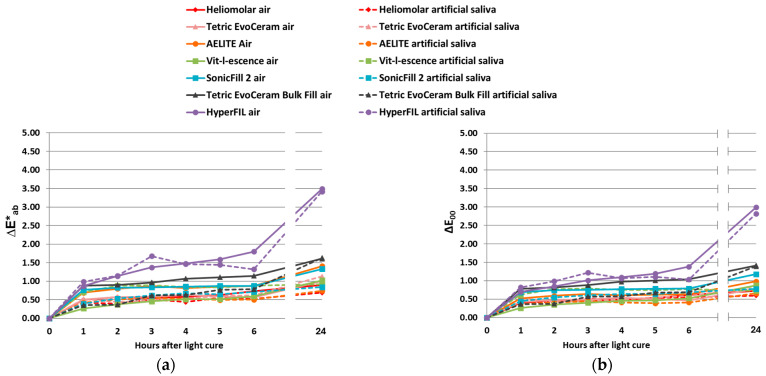
Mean values of ∆E of RCs after storage for 1, 2, 3, 4, 5, 6 and 24 h after light curing are shown. *N* is 8 for each group. ∆E*_ab_ was calculated by the CIELab formula (**a**). ΔE_00_ was calculated by the CIEDE2000 formula (**b**). Solid lines represent storage in room air, while dashed lines represent storage in artificial saliva.

**Figure 6:**
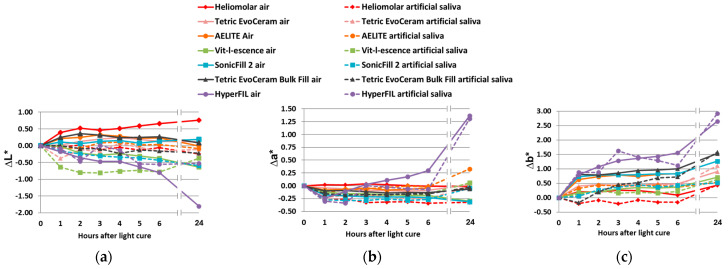
Mean values of ΔL* (**a**), Δa* (**b**) and Δb* (**c**) of RCs after storage for 1, 2, 3, 4, 5, 6 and 24 h are shown. *N* is 8 for each group. ∆L*, Δa* and Δb* were calculated by the CIELab formula. ∆L* is the difference in L*. ∆a* is the difference in a*. ∆b* is the difference in b*.

**Figure 7:**
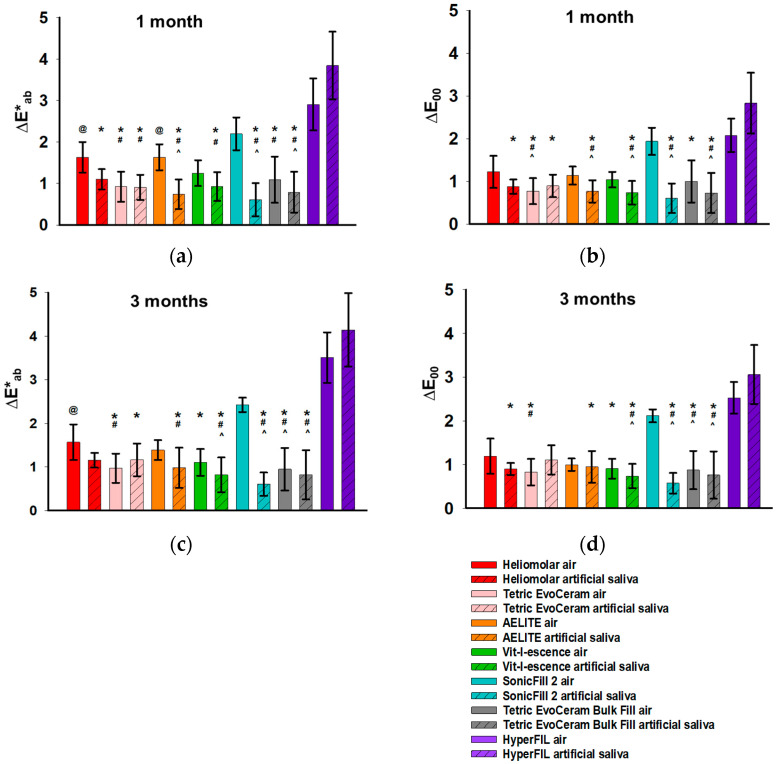
Color changes in RCs after aging for 1 month and 3 months are shown. ∆E was calculated by CIELab formula (**a**,**c**) and CIEDE2000 formula (**b**,**d**). *N* is 8 for each group. Bars show the mean and standard deviation. ANOVA on rank test was conducted. *, #, ^ and @ show significance vs. HyperFIL artificial saliva, HyperFIL air, SonicFill 2 air and SonicFill 2 artificial saliva, respectively. Solid bars represent aging in air, while striped bars represent aging in artificial saliva.

The samples were stored in either artificial saliva or room air after the measurements at “soon after light cure” were obtained. The mean ΔE values between soon after light curing and different time points in the first day are shown in Figure 5. During the first day of aging, the overall color changes ΔE*_ab_ (Figure 5a) and ΔE_00_ (Figure 5b) for all RCs except HyperFIL (Figure 5) were smaller than the 50:50% acceptability threshold (2.7 for ΔE*_ab_ and 1.8 for ΔE_00_), below which is considered clinically acceptable [19]. It was hard to determine whether or not such color change ΔE was mainly due to changes in L*, a* or b*, because there was not a clear pattern in the changes associated with L*, a* or b* (Figure 6). Note that the scales in Figure 6a–c are different to better show the differences between the groups comparatively. ΔE_00_ changed with time in the same pattern, again smaller in value compared to ΔE*_ab_ (Figure 5a,b). Storage conditions did not affect color change at 24 h but led to significant color changes after 1 month and 3 months of aging (Table 3). The CIELab and CIEDE2000 formulae resulted in the same statistical results for all three time points analyzed (1 day, 1 month and 3 months) (Table 3). In order to study the effect of the brand on color change after aging in more detail, a one-way ANOVA was performed on the ΔE values of all the RCs between soon after light curing and 1 and 3 months after light curing (Figure 7). Though ΔE*_ab_ (Figure 7a) and ΔE_00_ (Figure 7b) showed similar patterns, the CIEDE2000 formula resulted in smaller values than the CIELab formula, and the statistical significance was not always the same when calculated using different formulae. These results indicated that the two formulae were not just merely scaled values. The aging method affected the color of the RCs (Table 3). In most cases, if a significant difference occurred, those in artificial saliva showed better stability in color or a smaller ΔE (Figure 7). However, the dual-cure bulk-fill resin composite HyperFIL showed the opposite effect with aging method, in which those in room air tended to be more stable in color (Figure 7).

## 4. Discussion

Even though RCs with other photoinitiators have been studied, and some were shown to be good substitutes for CQ experimentally [12,20,21], currently, it appears there are no non-CQ dental RCs available in the U.S. market [3,8]. The fact that most currently available light-curing units on the market and in dental offices are optimized for curing CQ and may not be suitable for exciting alternative photoinitiators that absorb light at different wavelengths may contribute to the dominant use of CQ. The first and second generations of light-emitting diode (LED) light (single diode) have problems in curing photoinitiators that are sensitive to the shorter wavelengths of less than 420 nm of blue light. They only produce a narrow irradiation spectrum with longer wavelengths of light in the 450–470 nm range that cover the absorption spectrum of CQ [14]. The third-generation LED lights (polywave), however, are able to cure CQ (peak near 470 nm) and photoinitiators with an absorption spectrum below 430 nm [22]. The halogen light used in this study provides a broad irradiation spectrum, covering the absorption spectra of both CQ and TPO [22]. Another significant disadvantage of using photoinitiators with a shorter wavelength absorption peak is that they cause a lower depth of cure compared to CQ. The shorter wavelengths of light are scattered much more than the longer wavelengths of light, and as the thickness of the restoration increases, very few of these shorter wavelengths penetrate through the RCs and reach the bottom of the restoration [14]. Price et al. further suggested that unless they are very translucent, RCs that use predominantly alternative photoinitiators should be cured in small increments, by which the bottom of the RCs could be exposed to more of the shorter wavelength [14]. Depth of cure was not studied in the current study. Considering the factors discussed, it seems reasonable for manufacturers to keep CQ as a major component photoinitiator in their RCs before a perfect photoinitiator is found to totally substitute for CQ in the future. However, as mentioned, to reduce the yellowing effect of CQ, different approaches have been taken to select additional photoinitiators that can be added into the CQ/amine system to reduce the amount of CQ used. TPO appears to be a very successful approach. Among the five CQ+ RCs tested in this study, four of them contain TPO (the additional photoinitiator used in the other one (HyperFIL) is unknown), and three of them contain only TPO, besides CQ (Table 1). In contrast to CQ, which has a broad absorption spectrum with peak absorption around 470 nm [7,11,23,24], the absorption range of TPO is narrow and situated more toward the UV spectrum, 380–425 nm, with a peak at 400 nm [7,8,25]. TPO does not require the use of a co-initiator, and in this way, TPO is more efficient, as it reduces the intermediate steps required for radical production in the CQ/amine system [26]. Excitation of the TPO molecule produces two molecules with free radicals, which makes it more efficient for initiating the polymerization process compared to the CQ/amine system, which produces only one free radical per molecule [22,26]. The greater number of free radicals produced by TPO than CQ, however, may contribute to the inferior depth of cure in the RCs containing TPO. At similar concentrations, TPO absorbs many more photons than CQ, which, in turn, reduces the penetration of light through the RCs [26]. Nevertheless, TPO is considered to be a photoinitiator with high reactivity and curing efficiency [26], as TPO-based RCs have exhibited a higher degree of conversion [20,26] and rate of polymerization than those containing CQ with a tertiary amine [26]. Regardless of the many advantages of TPO, the major concern regarding this photoinitiator is toxicity. TPO shows higher cytotoxicity than CQ in specific experimental conditions [7,27]. However, the suspected pathway tested in these same studies, that is, through reactive oxygen/nitrogen species (ROS/RNS), cannot be verified [7,27]. Such cytotoxicity of TPO is concentration-dependent [27] and could be annulled by a thin dentin barrier [7]. While the use of TPO in RCs, especially when in high concentrations, raises concerns, following the manufacturer’s instructions and optimizing polymerization strategies can minimize the cytotoxic risk.

Speaking of color change upon polymerization, in the current study, except for the dual-cure HyperFIL, the RCs with CQ and additional photoinitiators showed a greater color change (Figure 3). The null hypothesis that the color change upon polymerization in RCs with additional photoinitiators is not different from that observed in RCs with CQ only is rejected. A possible reason for the greater color change in CQ+ may be that additional photoinitiators increased the degree of conversion. As mentioned, RCs with TPO show a greater degree of conversion compared to RCs with CQ only [20,26]. On the other hand, additional photoinitiators may also increase the speed of polymerization, because TPO increases the rate of polymerization [26]. Whether or not such a change in the polymerization rate absolutely affects the polymerization shrinkage stress remains controversial [23,28]. But the increased polymerization rate could have affected the color change upon polymerization due to differences in the degree of conversion at the time the color was measured in this study. As the conversion progresses, the polymeric network becomes denser, which impacts light transmission [29]. The color change after polymerization shown in Figure 3 was measured soon after light curing (within 10 s). The color change in the first day (Figure 5) reflected the ongoing polymerization process. When combined with statistical analysis (Table 3), it was determined that the type of photoinitiators used, not the storage conditions, had a significant effect on color change at 24 h.

The exact concentration of the total photoinitiators in the different RCs remains unknown. Although greater color changes were found in the RCs with additional photoinitiators in this study (Figure 3), it is possible that the RCs showing a greater color change included a greater concentration of photoinitiators, which has been shown to increase the degree of conversion within a certain threshold [23]. The concentration of the photoinitiators is critical. A good photoinitiator should have high absorption at low concentrations [24]. When the CQ concentration exceeds the critical level, the unreacted molecules will return to the ground state, which will result in yellow discoloration [30]. This may further attenuate the light and result in a reduced depth of cure [30]. Very high TPO concentrations will not increase the degree of conversion either, because TPO less than 1 wt% already results in a maximal degree of conversion [7]. On the other hand, when the concentration of photoinitiators is too low, the optimized rate of polymerization that results in optimal properties may not be able to be achieved. In cases where an additional photoinitiator is included in the RCs, the ratio of the amount of additional photoinitiators to CQ is important as well [14]. It is not possible to know the concentration of the photoinitiators or their ratios used in the commercial RCs because the information is considered to be proprietary. Experimental studies that control the concentrations of individual photoinitiators and their ratios are more definitive in ruling out the exact influence of the different photoinitiator systems. As mentioned, CQ requires the use of an amine as a co-initiator. The rate of polymerization and degree of conversion increase as the concentration of co-initiators increases [2]. In general, a higher amine content leads to improved polymer properties, but it is also correlated with a greater color change [31]. The different co-initiator formula used could also contribute to the difference in color change. Additional photoinitiators such as TPO may also react with the co-initiator of the CQ system and, therefore, speed up the polymerization process. Variables in the chemical composition of RCs besides photoinitiators, such as the types and concentrations of resin monomers and fillers, may also affect color changes during polymerization, because the degree of conversion can be affected by them. For instance, even though Tetric EvoCeram, which was categorized as CQ+, had a greater color change upon polymerization than the CQ-only RC Heliomolar, it had a smaller color change compared to the other two traditional CQ+ RCs, AELITE and Vit-l-escence (Figure 3a,b). According to the manufacturers, unlike AELITE and Vit-l-escence, Tetric EvoCeram does not contain any TEGDMA, a relatively smaller monomer that is able to diffuse more easily. This may contribute to its relatively smaller color change due to a lesser degree of conversion.

Changes in light scattering and absorption properties, like light reflectivity and translucency, may affect the color measurement [31]. In this study, the color measurement was based on reflective light. Translucency was not considered. Some manufacturers believe that the high translucency of RCs will result in a higher depth of cure [32]. A study by Kim et al. determined the translucency parameter values of a few commercial RCs and found that Tetric EvoCeram Bulk Fill had the highest translucency parameter, followed by SonicFill 2 and then Tetric EvoCeram (traditional RC). The result of this study is in agreement with their findings. The color change upon polymerization (Figure 3) in these three RCs (Tetric EvoCeram Bulk Fill > SonicFill 2 > Tetric EvoCeram) may be caused by the difference in the depth of cure, which is positively related to the translucency of the RCs.

Kim and Lee stated that light curing causes a characteristic chromatic shift toward the blue region of the color space that is away from yellow in RCs [33]. The results of the current study are in accord with this claim. In the current study, all the RCs tested became less yellow after polymerization (Figure 4c,f). When color matching clinically, these results suggest that it would be beneficial to choose an initial color that is slightly more yellow in color than the desired final color. Such a suggestion is consistent with a previous report [34]. In this way, the color shifting away from yellow after polymerization can be taken into consideration during color matching. And this suggestion applies to RCs with CQ only and those with CQ with additional photoinitiators. Alternatively, using custom shade tabs with already-polymerized RCs of the same brand would alleviate this need.

To study the effect of aging on color change, storage in room air and storage in artificial saliva were used as the aging methods. The effect of aging was studied after 1 month and 3 months of storage (Table 3 and Figure 7). The aging method affected the color change after both 1 and 3 months, while the type of photoinitiator did not (Table 3). Therefore, the null hypothesis that the color change upon aging in RCs with additional photoinitiators is not different from that observed in RCs with CQ only cannot be rejected. This study indicated that color change is not affected by the use of additional photoinitiators during the aging process. When differences in color change occurred, aging in air caused a greater color change than aging in artificial saliva, except in the case of HyperFIL (Figure 7). The oxidation of unreacted monomers and degradation due to dehydration with time (samples were incubated dry) may contribute to the greater color change after aging in air [35]. Even though the data within the first day up to 24 h were available (Figure 5 and Figure 6), the samples are not considered aged at these time points. Conflicting data exists in the literature about the effect of photoinitiators on color change after aging; though, to our knowledge, these studies are all on experimental formulations, not commercial RCs. Our data are in agreement with a previous study that showed that the type of photoinitiator did not interfere with the color stability due to aging [36]. But there is evidence also supporting better stability after aging in CQ-based RCs [37] and in TPO-based RCs [38]. Using commercial RCs, this study provides valuable data to the literature. Future studies on color change due to photoinitiators using various aging protocols such as thermocycling will be beneficial. With regard to visual thresholds and perceptibility and acceptability thresholds, variations in values exist in the literature [39]. Multiple factors, such as the method used, the observer’s gender and clinical expertise and the material under consideration, contribute to these variations [40,41,42]. The perceptibility threshold (PT) is the smallest color difference that can be detected by an observer. A 50:50% perceptibility threshold (50:50% PT) refers to a situation in which 50% of observers notice a difference in color while the other 50% notice no difference [39]. Correspondingly, a 50:50% acceptability threshold (50:50% AT) refers to the difference in color that is considered acceptable by 50% of observers [39]. Esthetically speaking, it should be ideal to achieve a color change that is smaller than 50:50% PT. But, in practice, color changes below 50:50% AT are generally considered to fulfill patients’ expectations of the esthetic outcome of dental restorations [39]. A 50:50% AT of 2.7 for ΔE*_ab_ and 1.8 for ΔE_00_ are well accepted in dentistry [19,39,43,44]. Though, as discussed, the statistical results showed that aging in air resulted in a greater color change in the majority of the RCs tested than aging in artificial saliva (Figure 7), such significance is less meaningful, because the changes in color due to aging in air and in artificial saliva were considered clinically acceptable for all the RCs tested in this study, except for HyperFIL according to both color-difference formulae and SonicFill 2 in air according to the CIEDE2000 formula (Figure 7). The internal color change in the resin matrix is suggested to be a reason for the discoloration of RCs upon aging [45]. On the contrary, the color change due to polymerization was greater than 50:50% AT for all the RCs tested, except for Heliomolar, a traditional RC with CQ only (Figure 3). These results suggested that unfavorable clinical esthetic outcomes due to color change are mainly caused by light curing rather than aging. Again, a prefabricated polymerized RC shade guide can facilitate clinical shade selection.

Several other factors are known to influence the color stability of RCs after aging. Translucency is a significant contributor. Highly translucent RCs should be used carefully because they present lower color stability [46]. Staining from the diet, such as from beverages [47] and smoking [48], has also been well documented as a major factor in color change during long-term clinical service. Furthermore, monomer type, filler content and filler size also affect RCs’ color stability [49]. While these aspects were not the primary focus of the present study, they are important considerations when interpreting esthetic performance and, therefore, should be further explored in future investigations.

Regardless of the level of color change upon polymerization and aging, the use of bulk-fill RCs is preferred by many dentists because it saves time [32]. Bulk-fill RCs are designed to be filled and cured in 4 mm thickness increments in a single step, and, therefore, they save time by skipping the time-consuming layering process [32]. When bulk-fill RCs have low hardness and elastic modulus, a surface cap layer is required to overcome the low wear resistance. The three bulk-fill RCs tested in this study were chosen because their manufacturers claim that they do not need to be covered with an outer layer of traditional RCs, because their elastic modulus and hardness are higher than those of other bulk-fill RCs [32]. Thus, the color stability of these bulk-fill RCs is an important issue with regard to their esthetic properties when used clinically. Tetric EvoCeram Bulk Fill contains CQ, TPO and Ivocerin^®^. The absorption peak of Ivocerin^®^ is around 418 nm [8]. In this study, Tetric EvoCeram Bulk Fill showed a large color change with light curing (Figure 3c,d). This may reflect its high polymerization rate and degree of conversion. On the other hand, Tetric EvoCeram Bulk Fill showed good color stability that is comparable to that of the traditional RCs upon aging (Figure 7). This is consistent with a previous study that found that a germanium-derivative photoinitiator increased DC and color stability [50].

HyperFIL behaved differently from the other bulk-fill RCs. It showed a small color change upon polymerization (Figure 3) but a large color change upon aging (Figure 5 and Figure 7). HyperFIL is a dual-cure RC. At the time that light was applied, the polymerization process had already started, because, in order to produce smooth surfaces, a 5 kg weight was applied and kept on all the samples for three minutes before light curing and, in the case of HyperFIL, after mixing. The color change upon polymerization for these RCs in the current study was actually reflecting the color change upon light curing after three minutes of chemical curing. This explains the small value of ΔE shown in Figure 3c,d. However, HyperFIL also showed a substantial color change after aging that was above 50:50% AT according to both calculations at both 1 month and 3 months (Figure 7). Post-irradiation polymerization has been assumed to happen in cured RCs, as observed by a change in hardness over a period of time, which could be on the scale of minutes to months [20]. This theory may explain the substantial color change in HyperFIL upon aging. This dual-cure resin composite may experience a longer post-irradiation polymerization.

Using both the CIEDE2000 and CIELab formulae, the color change was calculated in the current study. The values are comparable in value, and ΔE_00_ is always smaller than ΔE*_ab_ (Figure 3, Figure 5 and Figure 7); though, as discussed, they are not just merely scaled values. This study, therefore, partially agreed with the previous report by Lee that supported the interchangeable use of the two formulae [51]. Both the CIELab and CIEDE2000 formulae are well accepted in the field of dentistry. CIELab has a longer history of use, while the CIEDE2000 formula corrects the nonuniformity of the CIELab color space, especially for small color differences [52]. Despite the equation being more complicated, the CIEDE2000 formula has been shown to provide a better fit for evaluating color differences in dental ceramics [52,53] and reflects the color differences in RCs as perceived by the human eye better than the CIELab formula [42]. The current results are in accordance with this claim. In this study, even though the values are comparable, the two formulae do not always lead to exactly the same statistical results, with Figure 7 as an example. ΔE_00_ results in more values that are greater than the 50:50% AT compared to ΔE*_ab_ (Figure 7). However, in addition to color change (ΔE), CIELab calculations also provide ΔL*, Δa* and Δb* values easily, which provide more detailed information on the lightness/darkness, changes toward red/green and changes toward yellow/blue, respectively (Figure 4 and Figure 6). By reporting color change as calculated by both the CIELab and CIEDE2000 formulae, this study provides valuable data that are beneficial for comparisons with previous and future studies.

This study has a few limitations. (1) Halogen light was used due to its wide spectrum of wavelengths. However, this light is not broadly used in dental practices nowadays. Frequently used curing lights with higher intensity, like multispectral LED lights, should be used in future studies when the photoinitiators investigated are known. (2) A thin, clear plastic film was introduced during sample preparation in order to create a smooth surface. Though the L*, a* and b* values calculated with and without films for each sample were carefully compared to make sure they were similar, the use of the film may introduce an artifact that could bias the results. (3) A 5 kg weight was applied on the uncured RCs for 3 min to achieve a smooth surface. But it, meanwhile, introduced an uncontrolled variable by allowing the extra chemical curing of the dual-cure RC HyperFIL that explained its outlier results. (4) While color change upon polymerization and aging was calculated by two formulae in this study, translucency was not investigated. Future studies on the translucency of these RCs are needed to complete the color profile of these materials. (5) Related properties like depth of cure and degree of conversion are not investigated. Future studies on such related properties will be indispensable. (6) By using phosphate-buffered-saline-based artificial saliva, this study avoided potential color alterations due to the use of certain organic components. However, this non-organic formulation oversimplified the oral environment and, therefore, may reduce ecological validity by underestimating the color change compared to organic formulations. (7) Aging was studied only in air and artificial saliva, while more clinically relevant storage conditions, such as thermocycling, staining with liquids or smoke, were not studied. Future studies on aging under these conditions will be beneficial. (8) Three time points were studied in the aging study. Considering the exploratory nature of time in this study, a regular ANOVA, instead of a repeated-measures or mixed-effect model, was used. But the approach, together with the comparisons of multiple other factors, such as traditional/bulk-fill and brands, poses a limitation. Future studies focusing on the effect of time on the color stability of these RCs are needed for each specific aging condition. (9) To our knowledge, the color stability during the lifetime of the RCs tested is unknown, and it was not explored in the current study as well. Future studies on this will be beneficial.

## 5. Conclusions

Color changes upon polymerization and aging in air and artificial saliva were tested for seven commercial RCs with either CQ only or CQ and additional photoinitiators. RCs with CQ+ showed a greater color change upon polymerization, not taking into consideration the dual-cure resin composite HyperFIL, whose curing process included a 3 min gap between the start of light and chemical curing under the experimental conditions. The aging method, rather than whether or not additional photoinitiator(s) were added to CQ, had an effect on the color change in the RCs tested after aging of up to 3 months. But the color changes after aging for 1 and 3 months were considered clinically acceptable by at least one calculating method for all RCs tested except HyperFIL. These results also suggest that bulk-fill commercial RCs, such as Tetric EvoCeram, can have a comparable color stability upon aging to traditional commercial RCs.

## Figures and Tables

**Table 1:** Summary of the resin composites tested and the photoinitiators used.

Resin Composites	Manufacturer/Shade	Photoinitiator	Reference
Heliomolar	Ivoclar ^a^/A2	Traditional CQ	[13]
Tetric EvoCeram	Ivoclar ^a^/A2	Traditional CQ + TPO	[5]
AELITE	Bisco ^b^/A2	Traditional CQ + TPO	[14]
Vit-l-escence	Ultradent ^c^/A2	Traditional CQ + TPO	[4]
SonicFill 2	Kerr ^d^/A2	Bulk-fill CQ	[15]
Tetric EvoCeram Bulk Fill	Ivoclar ^a^/(IVA/Universal A)	Bulk-fill CQ + Ivocerin + TPO	[15]
HyperFIL	Parkell ^e^/Universal (A2/B2)	Bulk-fill dual-cure CQ + unknown	Manufacturer

CQ = camphorquinone; TPO = trimethylbenzoyl diphenylphosphine oxide; ^a^: Amherst, NY, USA; ^b^: Schaumburg, IL, USA; ^c^: South Jordan, UT, USA; ^d^: Brea, CA, USA; ^e^: Edgewood, NY, USA.

**Table 2:** L*, a* and b* of uncured resin composites tested.

Resin Composites/Shade	Group	Aging Method	Before Light (*n* = 8)					
			L*		a*		b*	
			Mean	SD	Mean	SD	Mean	SD
Heliomolar/A2	Traditional CQ	Artificial Saliva	61.97	1.05	−1.43	0.25	14.29	0.70
		Air	62.26	1.29	−1.50	0.42	14.44	0.65
Tetric EvoCeram/A2	Traditional CQ+	Artificial Saliva	56.64	0.70	−0.50	0.05	10.23	0.24
		Air	56.91	1.05	−0.54	0.08	10.07	0.28
AELITE/A2	Traditional CQ+	Artificial Saliva	59.82	0.67	0.78	0.10	10.09	0.47
		Air	59.60	0.62	0.79	0.11	10.71	0.20
Vit-l-escence/A2	Traditional CQ+	Artificial Saliva	66.98	0.99	−0.10	0.13	13.31	0.39
		Air	66.21	0.93	−0.06	0.11	13.51	0.16
SonicFill Bulk/A2	Bulk CQ	Artificial Saliva	58.46	0.43	−0.96	0.06	8.65	0.24
		Air	57.84	0.92	−0.91	0.09	7.89	0.36
Tetric EvoCeram Bulk/IVA/Universal A	Bulk CQ+	Artificial Saliva	53.19	0.42	−4.14	0.09	11.64	0.37
		Air	53.89	0.84	−4.41	0.17	11.90	0.54
HyperFIL Bulk/Universal (A2/B2)	Bulk CQ+	Artificial Saliva	60.26	1.37	0.30	0.51	11.41	1.17
		Air	58.74	0.58	0.48	0.17	11.57	0.48

**Table 3:** Two-way ANOVA on color change in RCs at 1 day, 1 month and 3 months.

Source of Variation/*p*	ΔE*_ab_			ΔE_00_		
	1 d	1 m	3 m	1 d	1 m	3 m
photoinitiator (CQ only or CQ+)	# <0.001	0.566	0.519	# <0.001	0.801	0.625
aging method (air or artificial saliva)	0.186	# 0.004	# 0.016	0.217	# 0.004	# 0.012
photoinitiator × aging	0.56	# 0.021	# 0.015	0.646	# 0.005	# 0.002

∆E*_ab_ was calculated by the CIELab formula. ∆E_00_ was calculated by the CIEDE2000 formula. *p*: *p* value. # denotes significance.

## Data Availability

The original contributions presented in this study are included in the article. Further inquiries can be directed to the corresponding authors.

## Abbreviations

The following abbreviations are used in this manuscript:

CQ	Camphorquinone
CQ+	CQ and additional photoinitiator(s)
TPO	Trimethylbenzoyl diphenylphosphine oxide
RCs	Resin composites
ΔE	Color change

## References

[1] Ferracane J.L. (2024). A Historical Perspective on Dental Composite Restorative Materials. J. Funct. Biomater..

[2] Bittencourt B.F., Dominguez J.A., Farago P.V., Pinheiro L.A., Gomes O.M. (2014). Alternative coinitiators applicable to photocurable resin composites. Oral Health Dent. Manag..

[3] Kowalska A., Sokolowski J., Gozdek T., Krasowski M., Kopacz K., Bociong K. (2021). The Influence of Various Photoinitiators on the Properties of Commercial Dental Composites. Polymers.

[4] Santini A., Miletic V., Swift M.D., Bradley M. (2012). Degree of conversion and microhardness of TPO-containing resin-based composites cured by polywave and monowave LED units. J. Dent..

[5] Palin W.M., Senyilmaz D.P., Marquis P.M., Shortall A.C. (2008). Cure width potential for MOD resin composite molar restorations. Dent. Mater..

[6] Rueggeberg F.A., Giannini M., Arrais C.A.G., Price R.B.T. (2017). Light curing in dentistry and clinical implications: A literature review. Braz. Oral Res..

[7] Van Landuyt K.L., Krifka S., Hiller K.A., Bolay C., Waha C., Van Meerbeek B., Schmalz G., Schweikl H. (2015). Evaluation of cell responses toward adhesives with different photoinitiating systems. Dent. Mater..

[8] Kowalska A., Sokolowski J., Bociong K. (2021). The Photoinitiators Used in Resin Based Dental Composite—A Review and Future Perspectives. Polymers.

[9] Kowalska A., Sokolowski J., Szynkowska-Jozwik M.I., Gozdek T., Kopacz K., Bociong K. (2022). Can TPO as Photoinitiator Replace “Golden Mean” Camphorquinone and Tertiary Amines in Dental Composites? Testing Experimental Composites Containing Different Concentration of Diphenyl(2,4,6-trimethylbenzoyl)phosphine Oxide. Int. J. Mol. Sci..

[10] Porto I.C., Soares L.E., Martin A.A., Cavalli V., Liporoni P.C. (2010). Influence of the photoinitiator system and light photoactivation units on the degree of conversion of dental composites. Braz. Oral Res..

[11] Sim J.S., Seol H.J., Park J.K., Garcia-Godoy F., Kim H.I., Kwon Y.H. (2012). Interaction of LED light with coinitiator-containing composite resins: Effect of dual peaks. J. Dent..

[12] de Oliveira D., Rocha M.G., Correa I.C., Correr A.B., Ferracane J.L., Sinhoreti M.A.C. (2016). The effect of combining photoinitiator systems on the color and curing profile of resin-based composites. Dent. Mater..

[13] Guiraldo R.D., Consani S., Consani R.L., Berger S.B., Mendes W.B., Sinhoreti M.A., Correr-Sobrinho L. (2010). Comparison of silorane and methacrylate-based composite resins on the curing light transmission. Braz. Dent. J..

[14] Price R.B., Felix C.A. (2009). Effect of delivering light in specific narrow bandwidths from 394 to 515nm on the micro-hardness of resin composites. Dent. Mater..

[15] Rocha M.G., de Oliveira D., Correa I.C., Correr-Sobrinho L., Sinhoreti M., Ferracane J.L., Correr A.B. (2017). Light-emitting Diode Beam Profile and Spectral Output Influence on the Degree of Conversion of Bulk Fill Composites. Oper. Dent..

[16] Balachandran J., Vani K., Alagarsamy V., J P., Sabarathinam J. (2024). Analyzing the Bioactivity of a Novel Bone Cement: An In Vitro Study. Cureus.

[17] Pecho O.E., Ghinea R., Alessandretti R., Perez M.M., Della Bona A. (2016). Visual and instrumental shade matching using CIELAB and CIEDE2000 color difference formulas. Dent. Mater..

[18] Durand L.B., Ruiz-Lopez J., Perez B.G., Ionescu A.M., Carrillo-Perez F., Ghinea R., Perez M.M. (2021). Color, lightness, chroma, hue, and translucency adjustment potential of resin composites using CIEDE2000 color difference formula. J. Esthet. Restor. Dent..

[19] Paravina R.D., Ghinea R., Herrera L.J., Bona A.D., Igiel C., Linninger M., Sakai M., Takahashi H., Tashkandi E., Perez Mdel M. (2015). Color difference thresholds in dentistry. J. Esthet. Restor. Dent..

[20] Miletic V., Santini A. (2012). Micro-Raman spectroscopic analysis of the degree of conversion of composite resins containing different initiators cured by polywave or monowave LED units. J. Dent..

[21] Ilie N., Hickel R. (2008). Can CQ be completely replaced by alternative initiators in dental adhesives?. Dent. Mater. J..

[22] Randolph L.D., Palin W.M., Bebelman S., Devaux J., Gallez B., Leloup G., Leprince J.G. (2014). Ultra-fast light-curing resin composite with increased conversion and reduced monomer elution. Dent. Mater..

[23] Schneider L.F., Pfeifer C.S., Consani S., Prahl S.A., Ferracane J.L. (2008). Influence of photoinitiator type on the rate of polymerization, degree of conversion, hardness and yellowing of dental resin composites. Dent. Mater..

[24] Schroeder W.F., Cook W.D., Vallo C.I. (2008). Photopolymerization of N,N-dimethylaminobenzyl alcohol as amine co-initiator for light-cured dental resins. Dent. Mater..

[25] Leprince J.G., Palin W.M., Vanacker J., Sabbagh J., Devaux J., Leloup G. (2014). Physico-mechanical characteristics of commercially available bulk-fill composites. J. Dent..

[26] Popal M., Volk J., Leyhausen G., Geurtsen W. (2018). Cytotoxic and genotoxic potential of the type I photoinitiators BAPO and TPO on human oral keratinocytes and V79 fibroblasts. Dent. Mater..

[27] Lu H., Stansbury J.W., Bowman C.N. (2005). Impact of curing protocol on conversion and shrinkage stress. J. Dent. Res..

[28] Pfeifer C.S., Shelton Z.R., Braga R.R., Windmoller D., Machado J.C., Stansbury J.W. (2011). Characterization of dimethacrylate polymeric networks: A study of the crosslinked structure formed by monomers used in dental composites. Eur. Polym. J..

[29] Stansbury J.W. (2000). Curing dental resins and composites by photopolymerization. J. Esthet. Dent..

[30] Ogunyinka A., Palin W.M., Shortall A.C., Marquis P.M. (2007). Photoinitiation chemistry affects light transmission and degree of conversion of curing experimental dental resin composites. Dent. Mater..

[31] Schneider L.F., Cavalcante L.M., Consani S., Ferracane J.L. (2009). Effect of co-initiator ratio on the polymer properties of experimental resin composites formulated with camphorquinone and phenyl-propanedione. Dent. Mater..

[32] Kim E.H., Jung K.H., Son S.A., Hur B., Kwon Y.H., Park J.K. (2015). Effect of resin thickness on the microhardness and optical properties of bulk-fill resin composites. Restor. Dent. Endod..

[33] Kim I.J., Lee Y.K. (2007). Changes in color and color parameters of dental resin composites after polymerization. J. Biomed. Mater. Res. B Appl. Biomater..

[34] Seghi R.R., Gritz M.D., Kim J. (1990). Colorimetric changes in composites resulting from visible-light-initiated polymerization. Dent. Mater..

[35] Lee Y.K., Yu B., Lim H.N., Lim J.I. (2011). Difference in the color stability of direct and indirect resin composites. J. Appl. Oral Sci..

[36] Silami F.D., Mundim F.M., Garcia Lda F., Sinhoreti M.A., Pires-de-Souza Fde C. (2013). Color stability of experimental composites containing different photoinitiators. J. Dent..

[37] de Oliveira D.C., Rocha M.G., Gatti A., Correr A.B., Ferracane J.L., Sinhoret M.A. (2015). Effect of different photoinitiators and reducing agents on cure efficiency and color stability of resin-based composites using different LED wavelengths. J. Dent..

[38] Albuquerque P.P., Moreira A.D., Moraes R.R., Cavalcante L.M., Schneider L.F. (2013). Color stability, conversion, water sorption and solubility of dental composites formulated with different photoinitiator systems. J. Dent..

[39] Paravina R.D., Perez M.M., Ghinea R. (2019). Acceptability and perceptibility thresholds in dentistry: A comprehensive review of clinical and research applications. J. Esthet. Restor. Dent..

[40] Milagres V., Teixeira M.L., Miranda M.E., Osorio Silva C.H., Ribeiro Pinto J.R. (2012). Effect of gender, experience, and value on color perception. Oper. Dent..

[41] Kurt M., Nemli S.K., Gungor M.B., Bal B.T., Ozturk E. (2024). Perceptibility and acceptability thresholds for color differences of light and dark maxillofacial skin replications. Vis. Res..

[42] Gomez-Polo C., Portillo Munoz M., Lorenzo Luengo M.C., Vicente P., Galindo P., Martin Casado A.M. (2016). Comparison of the CIELab and CIEDE2000 color difference formulas. J. Prosthet. Dent..

[43] Ragain J.C., Johnston W.M. (2001). Minimum color differences for discriminating mismatch between composite and tooth color. J. Esthet. Restor. Dent..

[44] Da Silva J.D., Park S.E., Weber H.P., Ishikawa-Nagai S. (2008). Clinical performance of a newly developed spectrophotometric system on tooth color reproduction. J. Prosthet. Dent..

[45] Celik E.U., Aladag A., Turkun L.S., Yilmaz G. (2011). Color changes of dental resin composites before and after polymerization and storage in water. J. Esthet. Restor. Dent..

[46] Salgado V.E., Rego G.F., Schneider L.F., Moraes R.R., Cavalcante L.M. (2018). Does translucency influence cure efficiency and color stability of resin-based composites?. Dent. Mater..

[47] Barutcigil C., Barutcigil K., Ozarslan M.M., Dundar A., Yilmaz B. (2018). Color of bulk-fill composite resin restorative materials. J. Esthet. Restor. Dent..

[48] Paolone G., Pavan F., Guglielmi P.C., Scotti N., Cantatore G., Vichi A. (2022). In vitro procedures for color stability evaluation of dental resin-based composites exposed to smoke: A scoping review. Dent. Mater. J..

[49] Alghazzawi T.F. (2024). The effects of monomer type, filler size, and filler content of three resin cements on the color stability of laminate veneers exposed to accelerated aging. Dent. Mater..

[50] Alkhudhairy F., Vohra F., Naseem M., Owais M.M., Amer A.H.B., Almutairi K.B. (2020). Color stability and degree of conversion of a novel dibenzoyl germanium derivative containing photo-polymerized resin luting cement. J. Appl. Biomater. Funct. Mater..

[51] Lee Y.K. (2005). Comparison of CIELAB Δ*E** and CIEDE2000 color-differences after polymerization and thermocycling of resin composites. Dent. Mater..

[52] Acar O., Yilmaz B., Altintas S.H., Chandrasekaran I., Johnston W.M. (2016). Color stainability of CAD/CAM and nanocomposite resin materials. J. Prosthet. Dent..

[53] Ghinea R., Perez M.M., Herrera L.J., Rivas M.J., Yebra A., Paravina R.D. (2010). Color difference thresholds in dental ceramics. J. Dent..

